# Supplementary material for: Further Developments towards a Minimal Potent Derivative of Human Relaxin-2
Source: Int J Mol Sci. 2023 Aug 11;24(16):12670. doi: 10.3390/ijms241612670 (PMC10454739; doi:10.3390/ijms241612670)

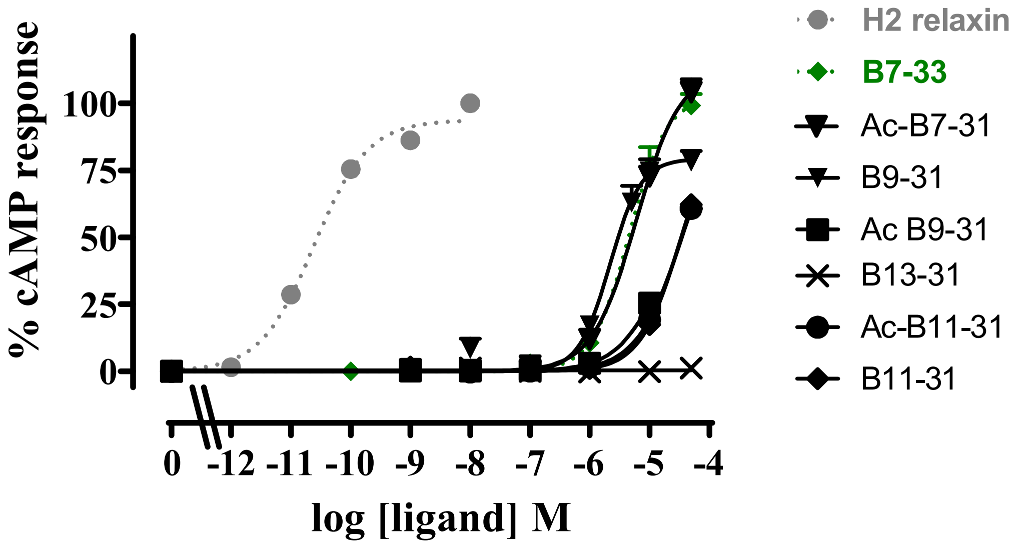

Figure S 1. cAMP response data for analogues presented in figure 3.

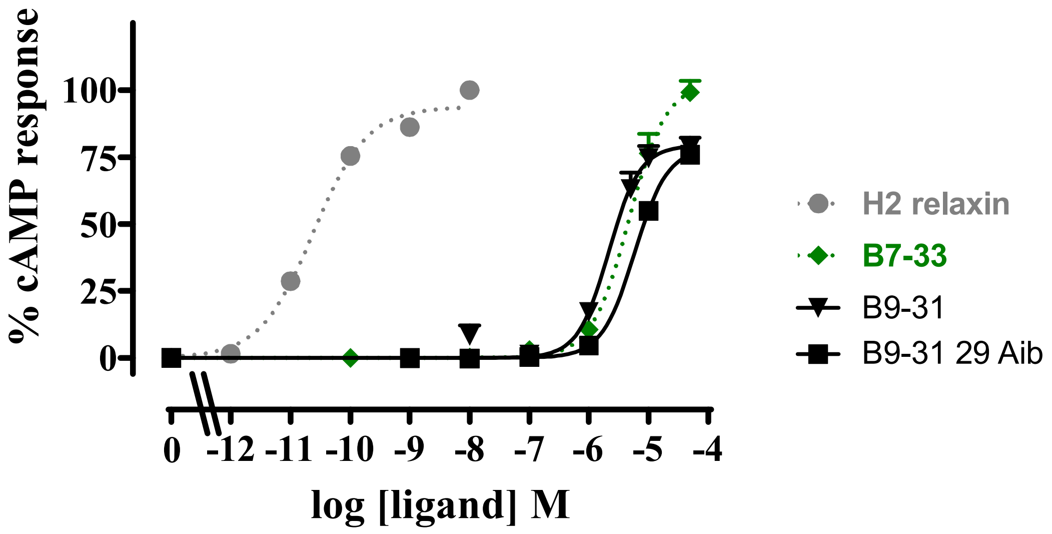

Figure S2. cAMP response data for analogues presented in Figure 4.

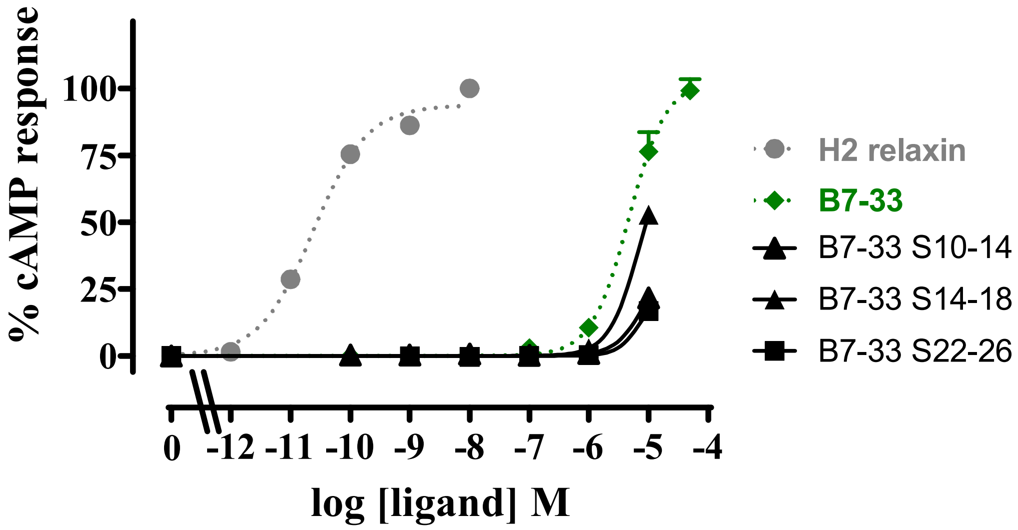

Figure S3 cAMP response data for analogues presented in figure 5.

Figure S4- HPLC and MALDI data for select peptides. Some of the papers presented in this paper are previously published in ref 32. Any excluded HPLC data and MS data is available on request.

a. B7-33

RP-HPLC carried out using the elution gradient: buffer B 20-50% in 30 minutes, t= 17.081

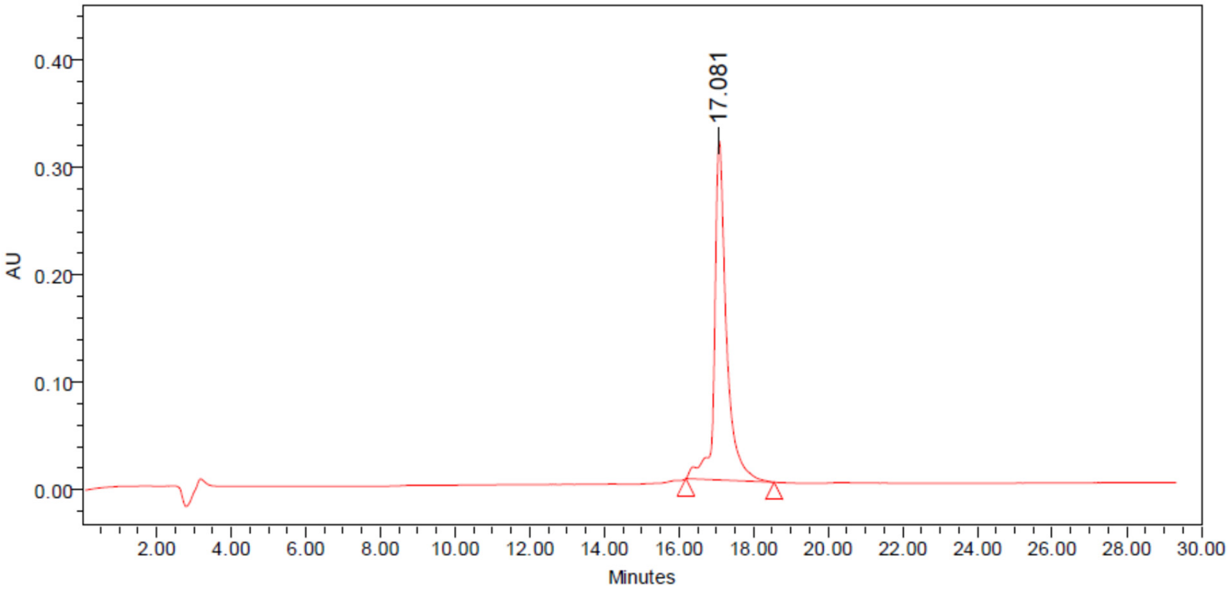

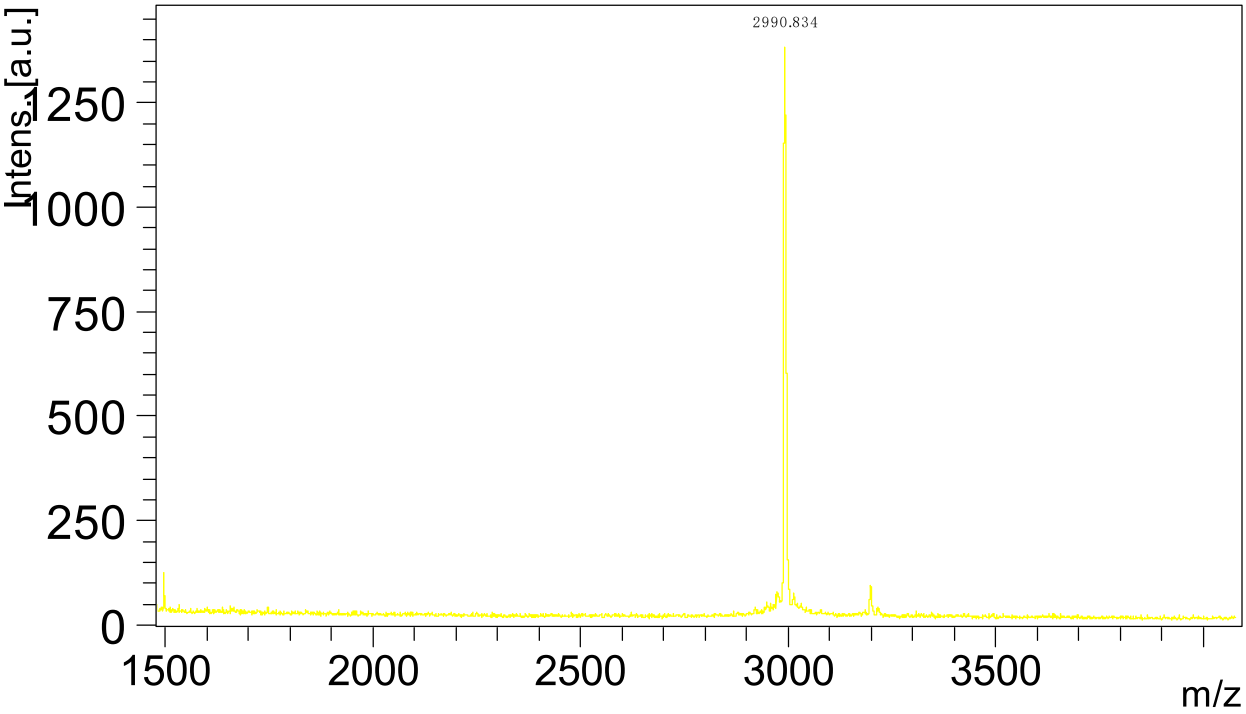

b. B7-31

RP-HPLC carried out using the elution gradient: buffer B 20-50% in 30 minutes, t= 16.008

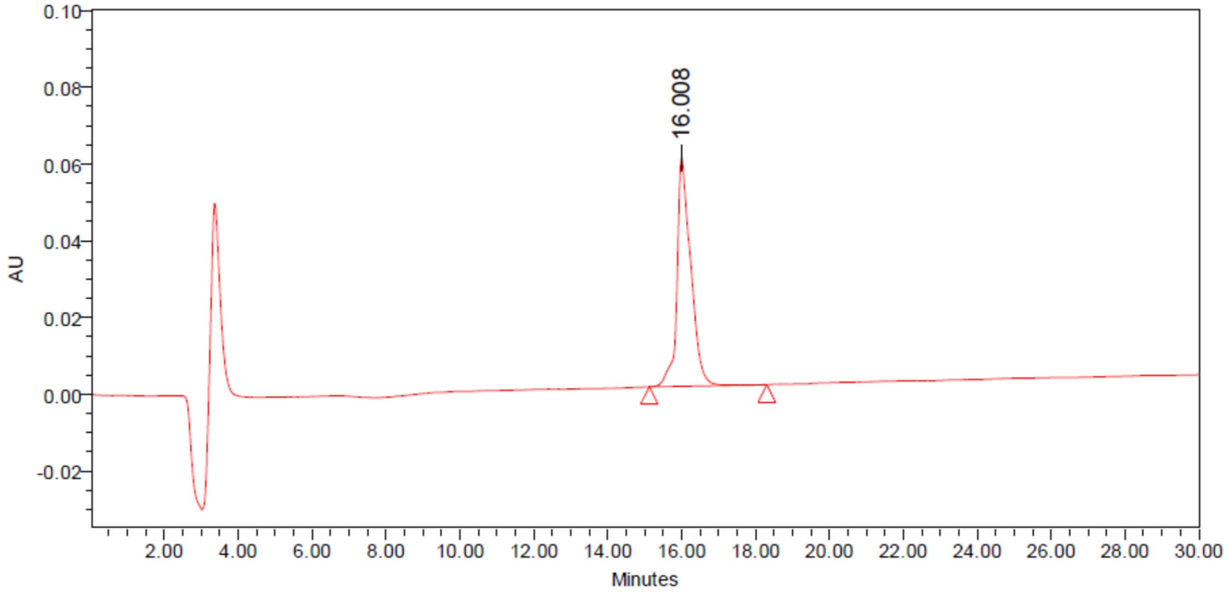

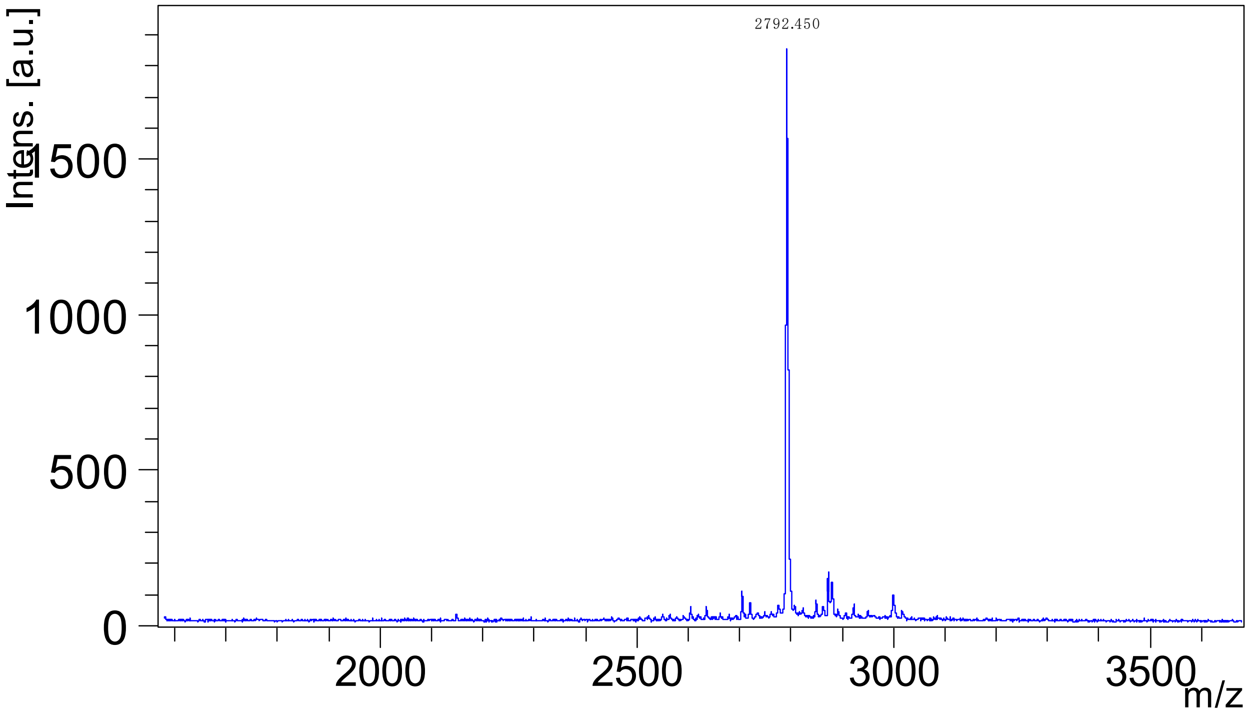

c. B9-31

RP-HPLC carried out using the elution gradient: buffer B 15-45% in 30 minutes, t= 17.831

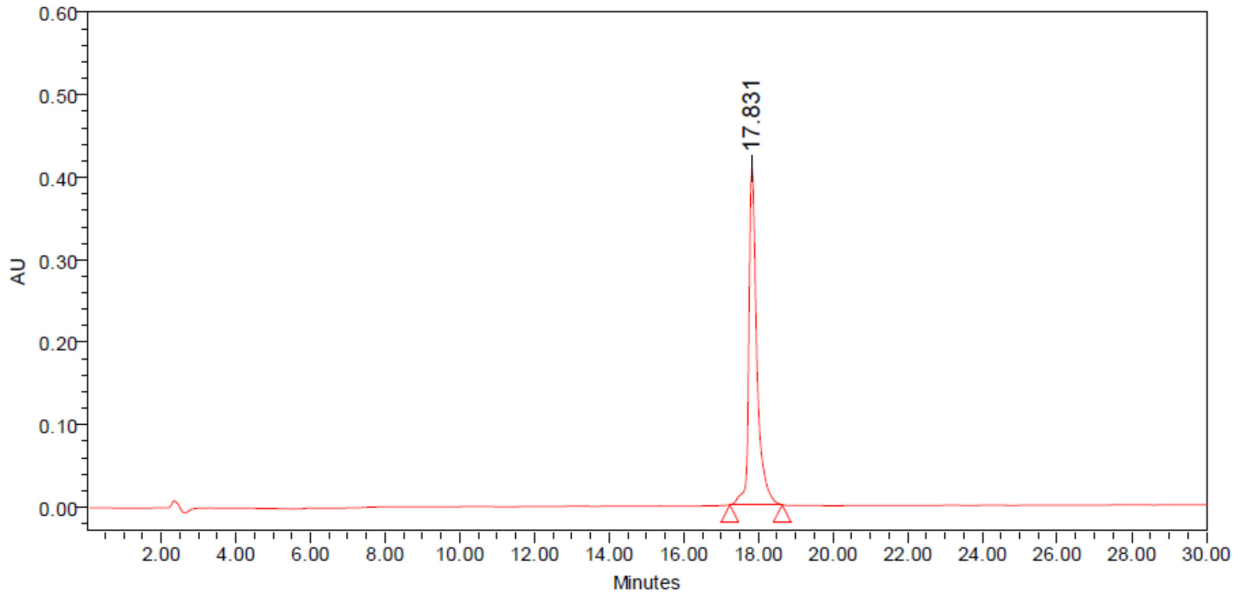

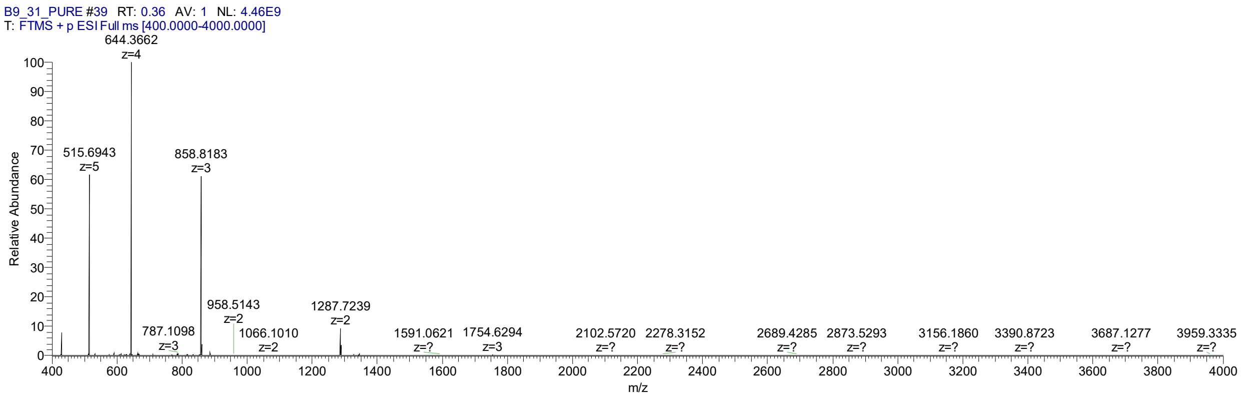

d. AcB9-31

RP-HPLC carried out using the elution gradient: buffer B 15-45% in 30 minutes, t= 19.336

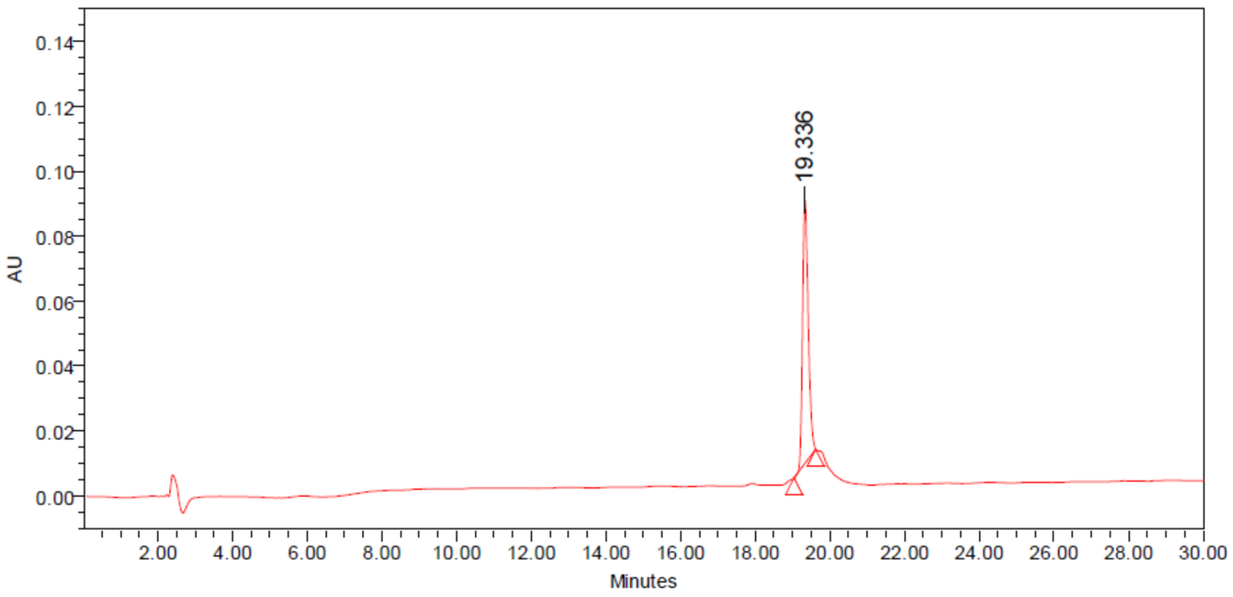

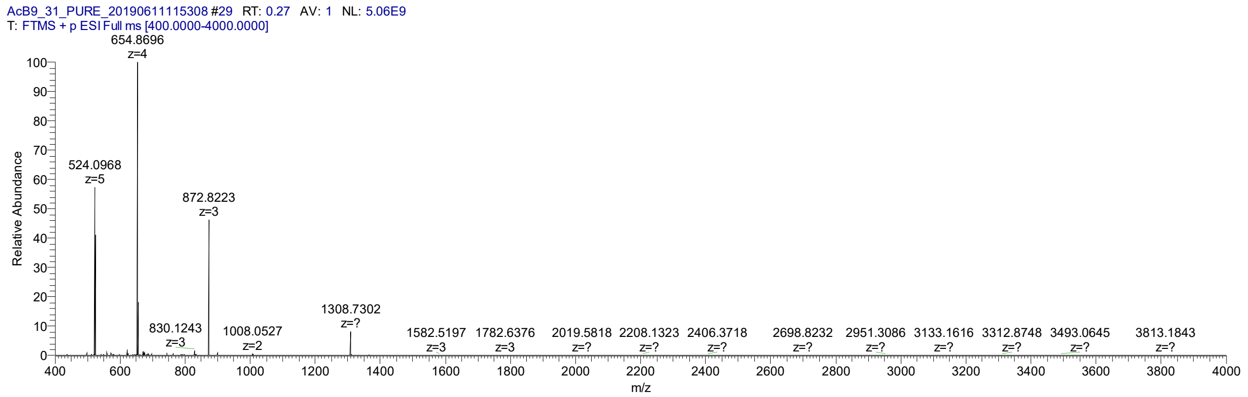

e. B11-31

RP-HPLC carried out using the elution gradient: buffer B 15-45% in 30 minutes, t= 17.818

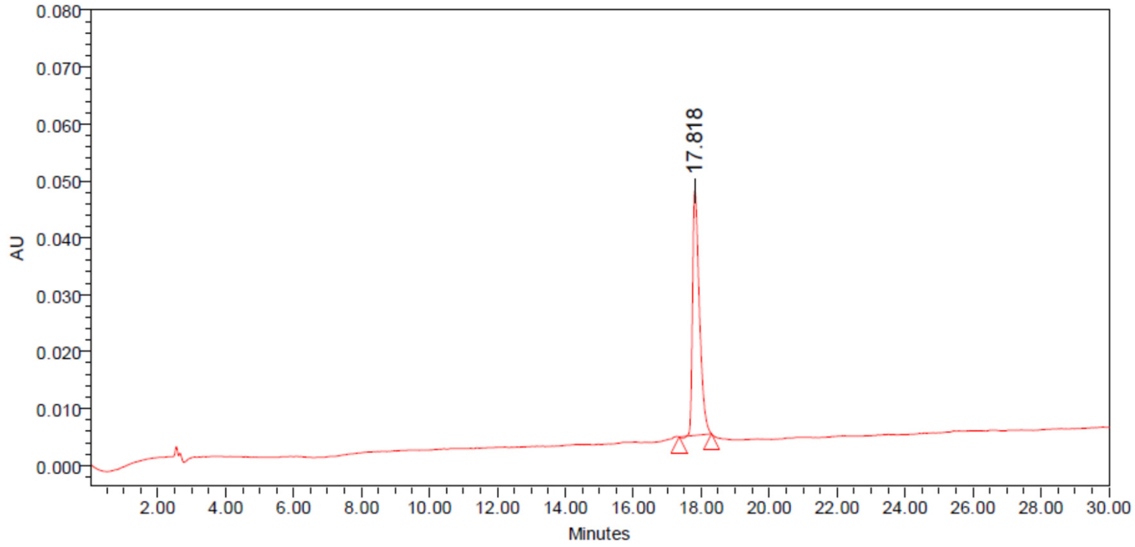

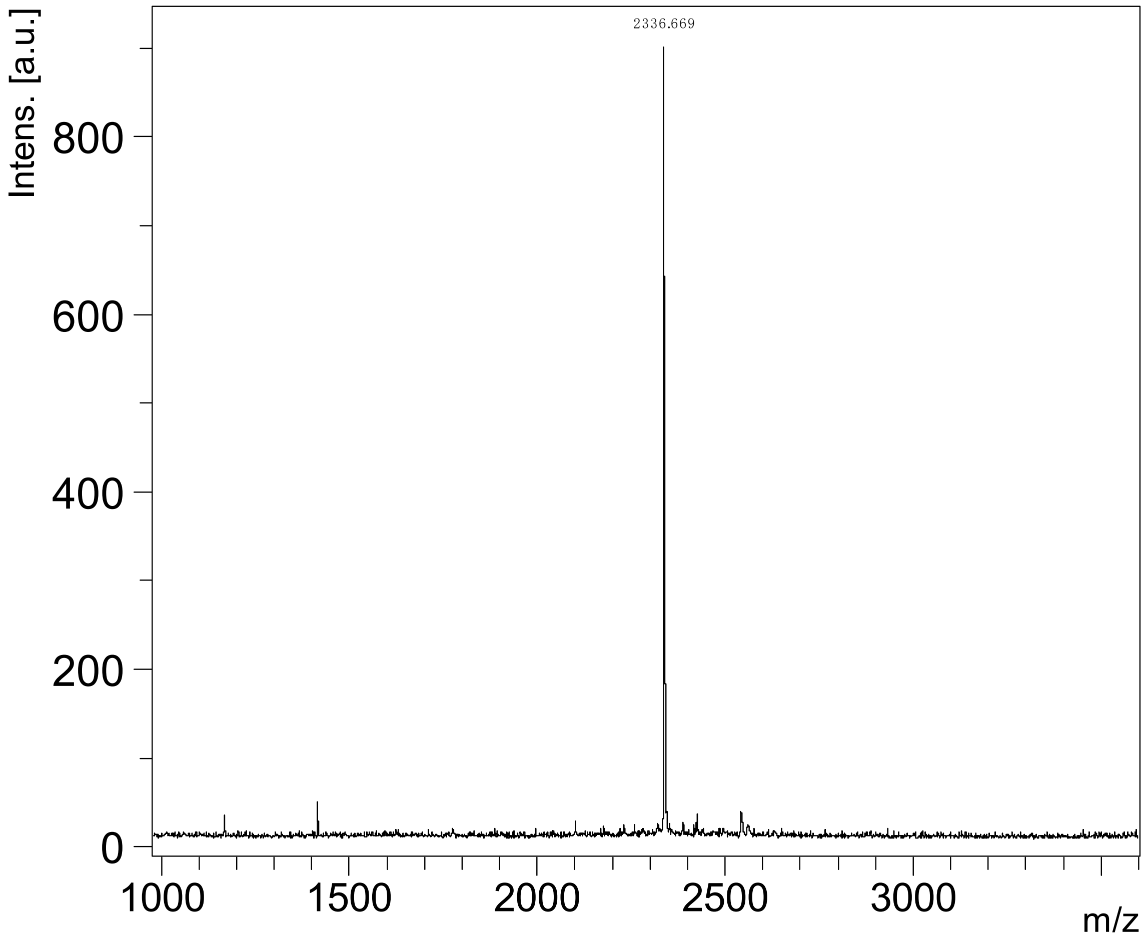

f. AcB11-31

RP-HPLC carried out using the elution gradient: buffer B 20-50% in 30 minutes, t= 14.841

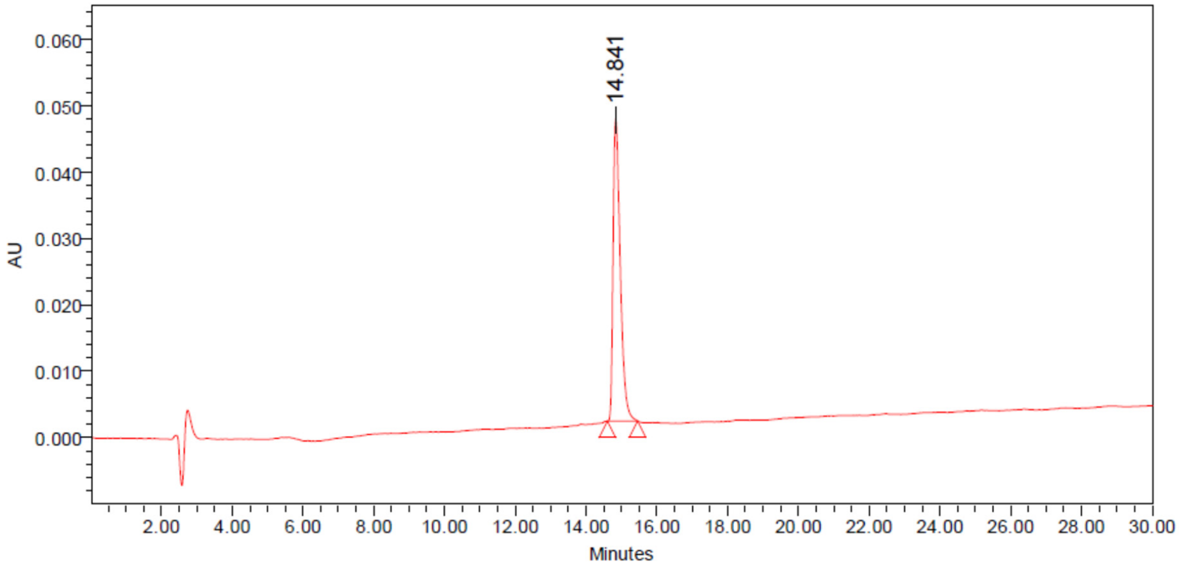

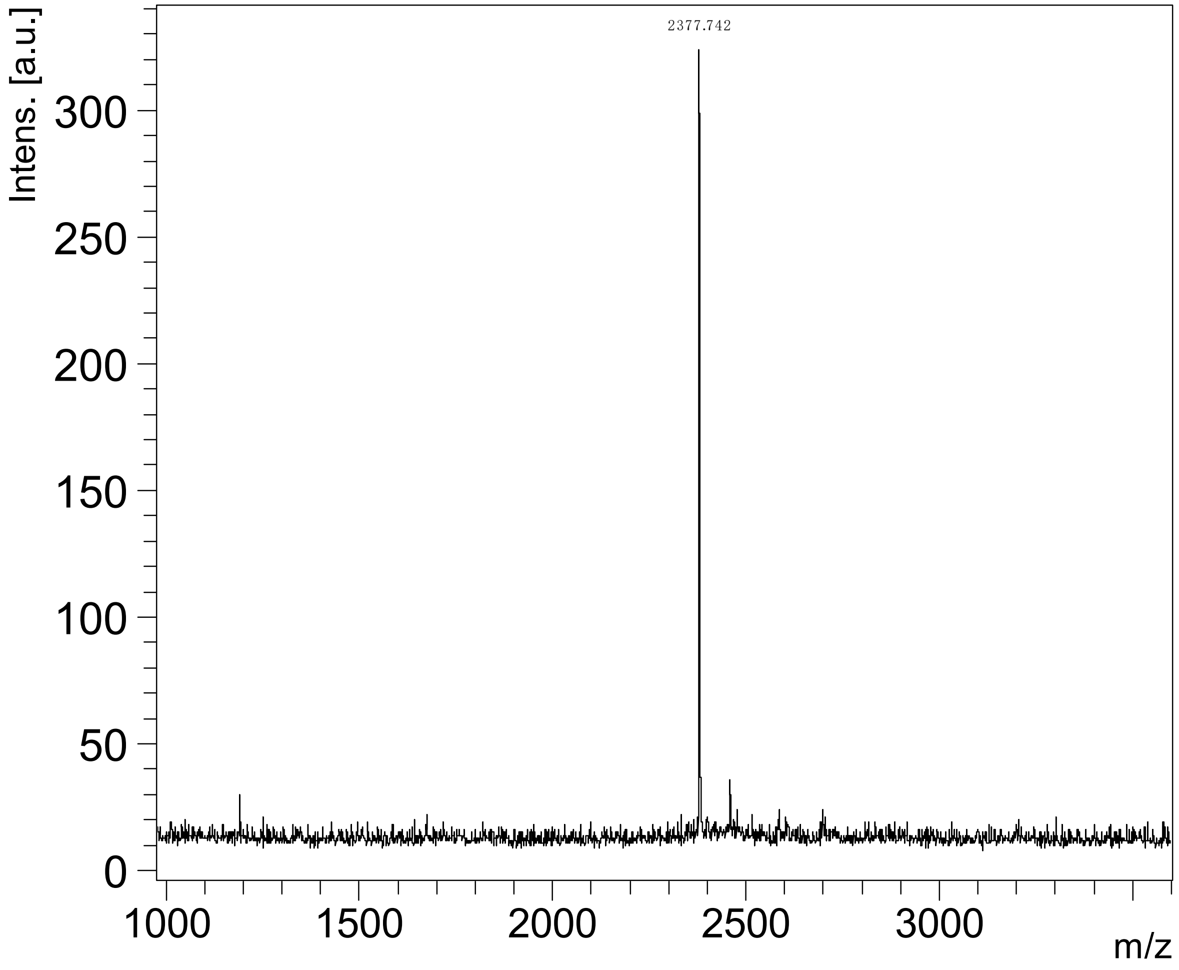

g. B13-31

RP-HPLC carried out using the elution gradient: buffer B 15-45% in 30 minutes, t= 17.203

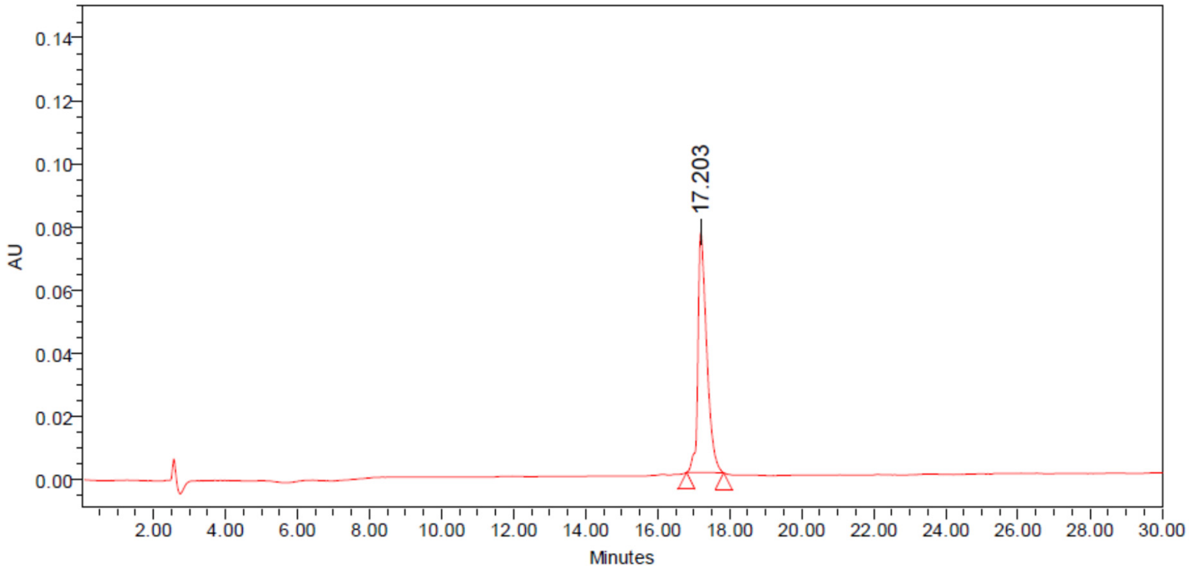

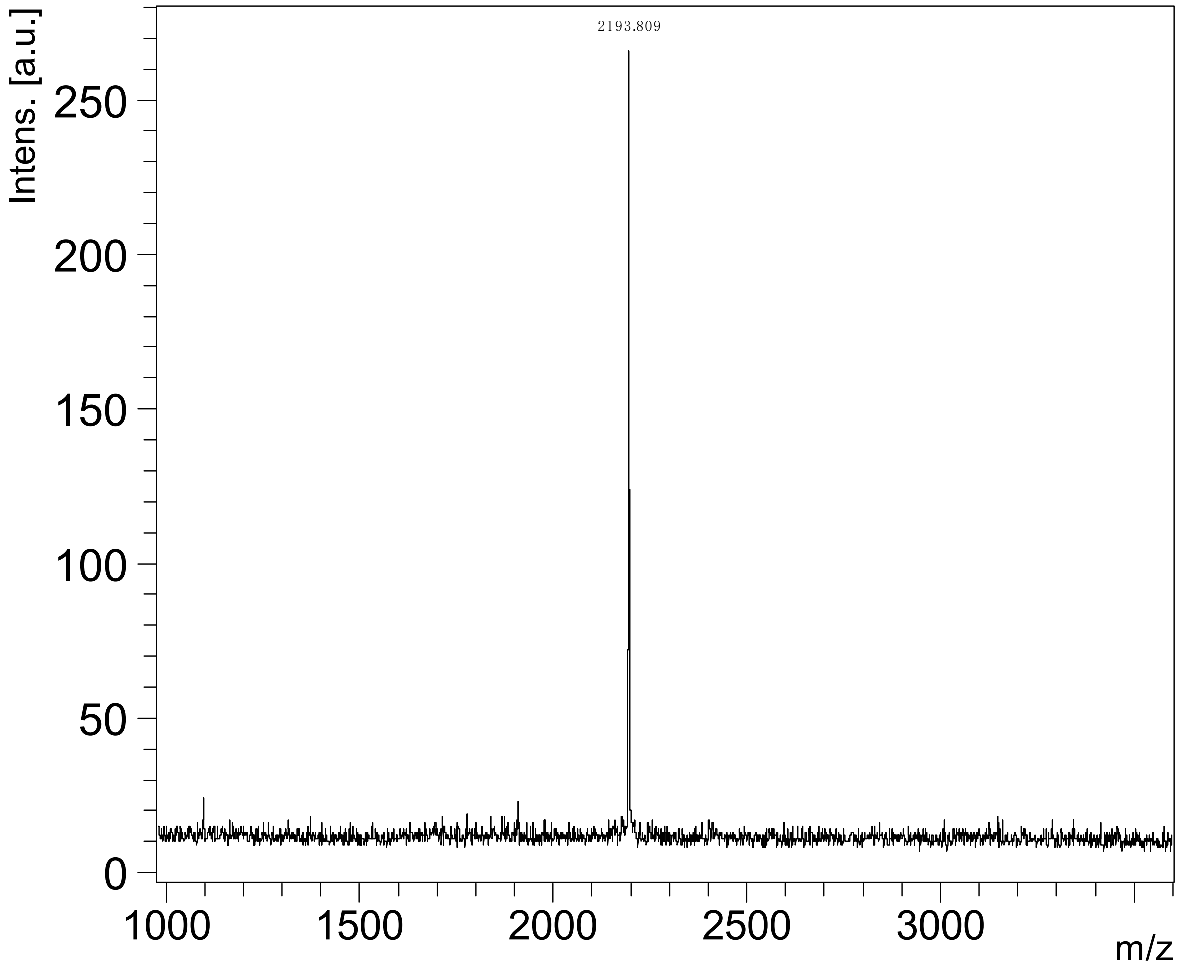

h. AcB13-31

RP-HPLC carried out using the elution gradient: buffer B 20-50% in 30 minutes, t= 14.841

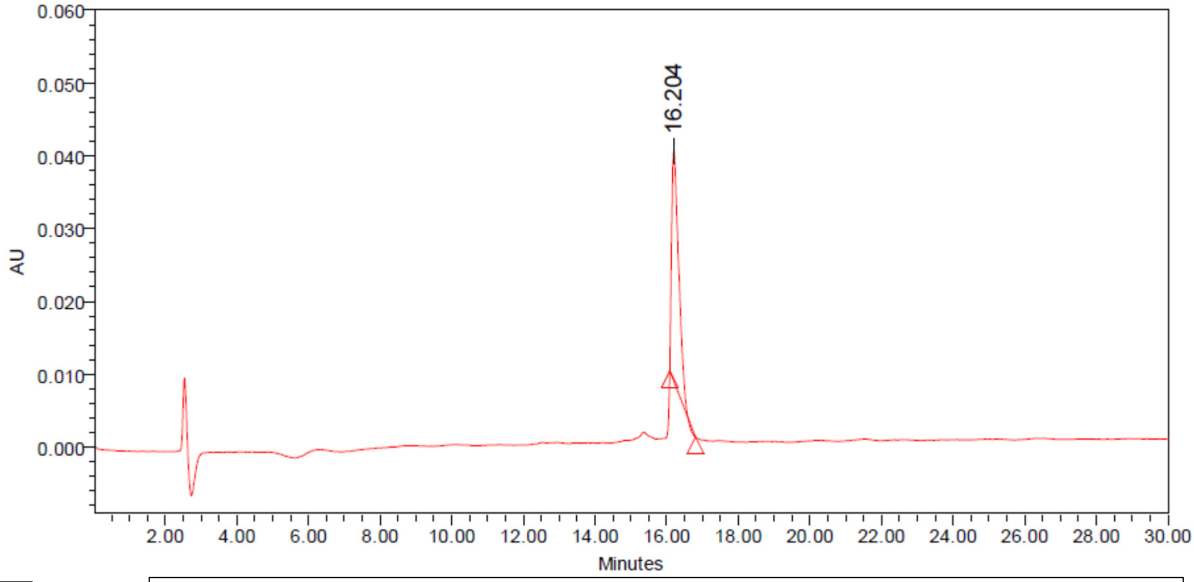

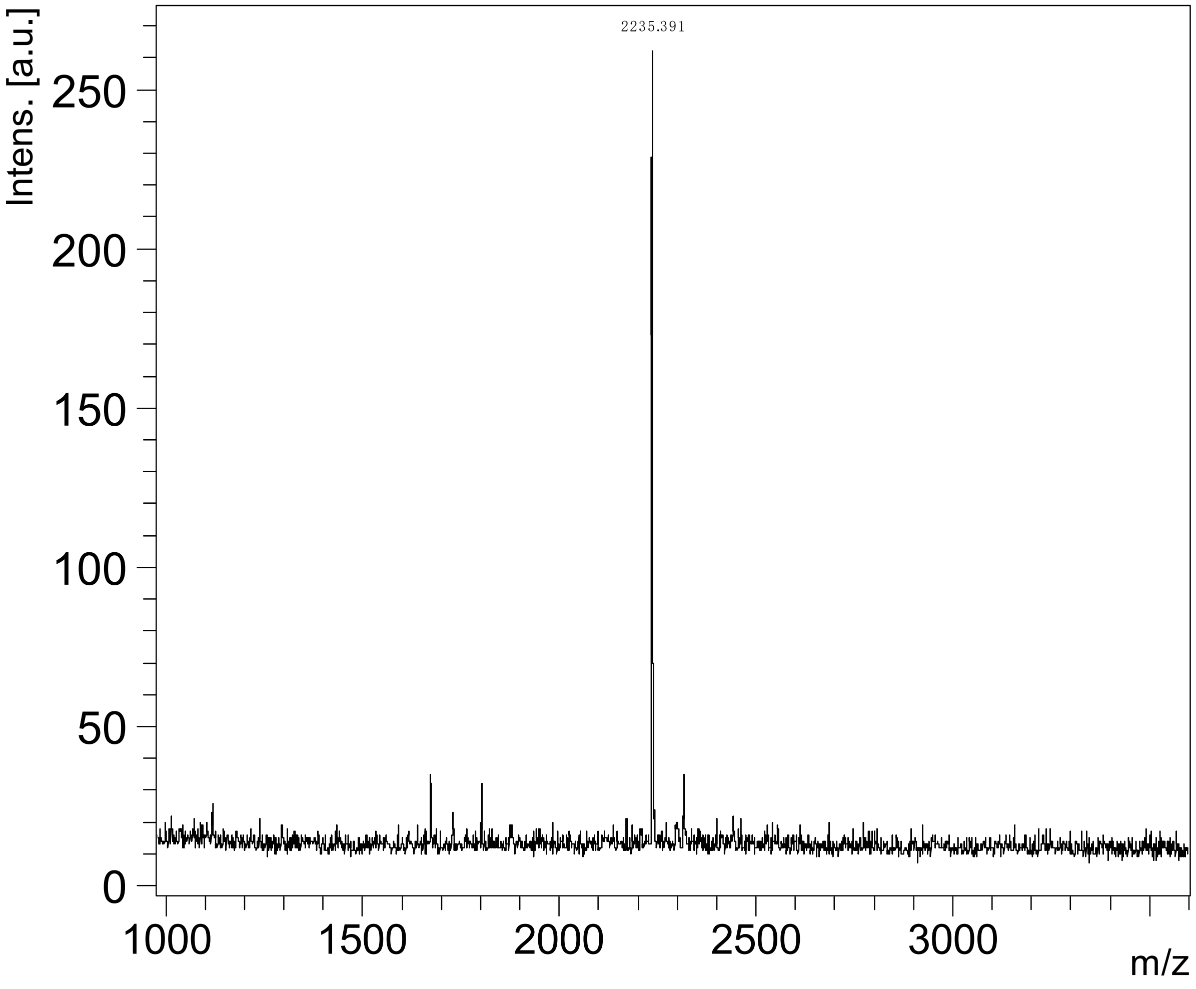

i. B9-31-29Aib

RP-HPLC carried out using the elution gradient: buffer B 20-50% in 30 minutes, t= 16.359

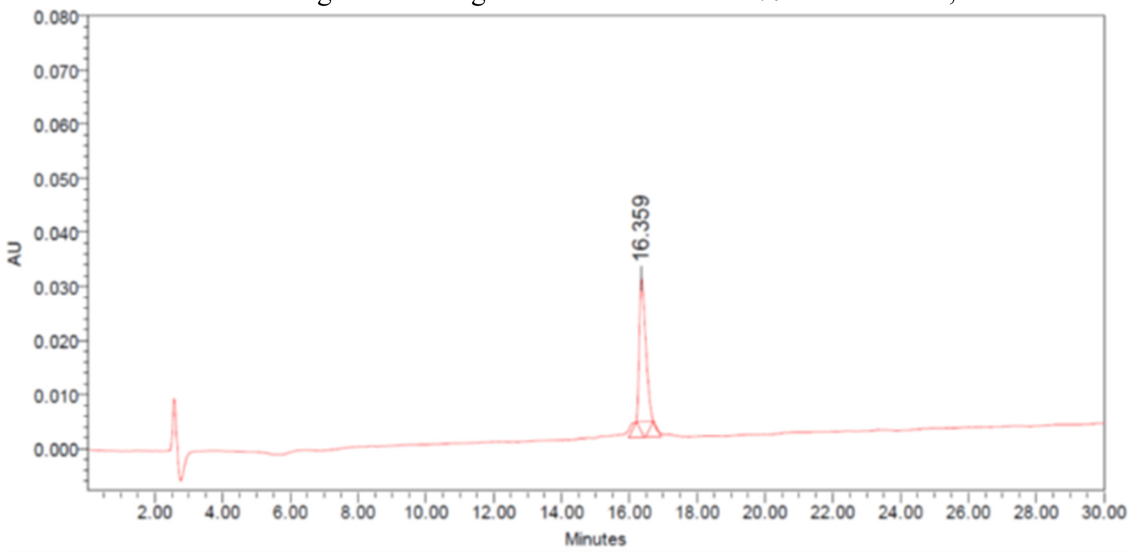

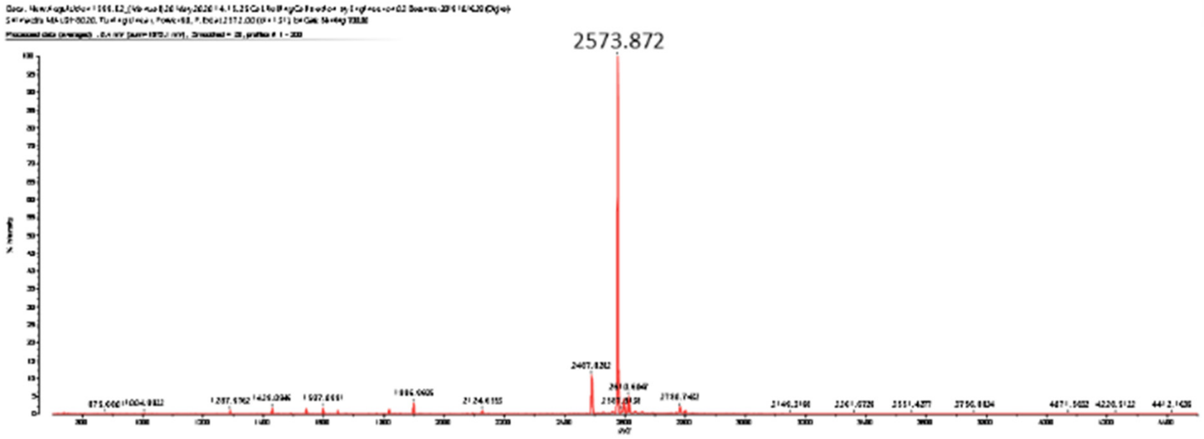

j. B9-31Aib 25/29

RP-HPLC carried out using the elution gradient: buffer B 20-50% in 30 minutes, t= 15.891.

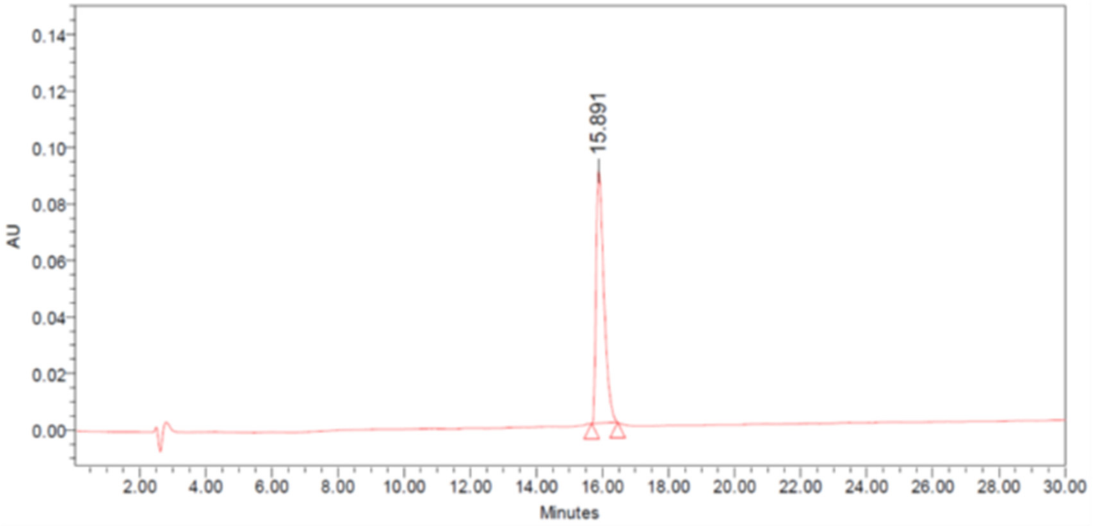

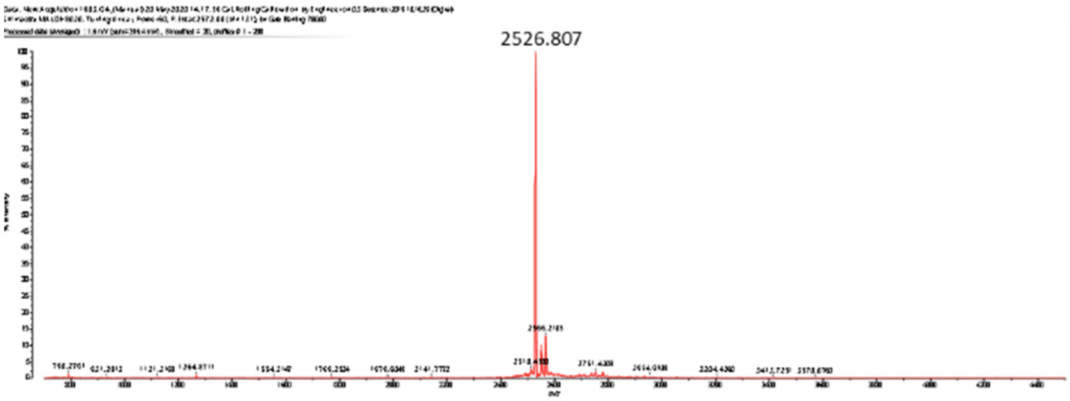

k. B9-31Aib 18/25/29

RP-HPLC carried out using the elution gradient: buffer B 20-50% in 30 minutes, t= 17.535.

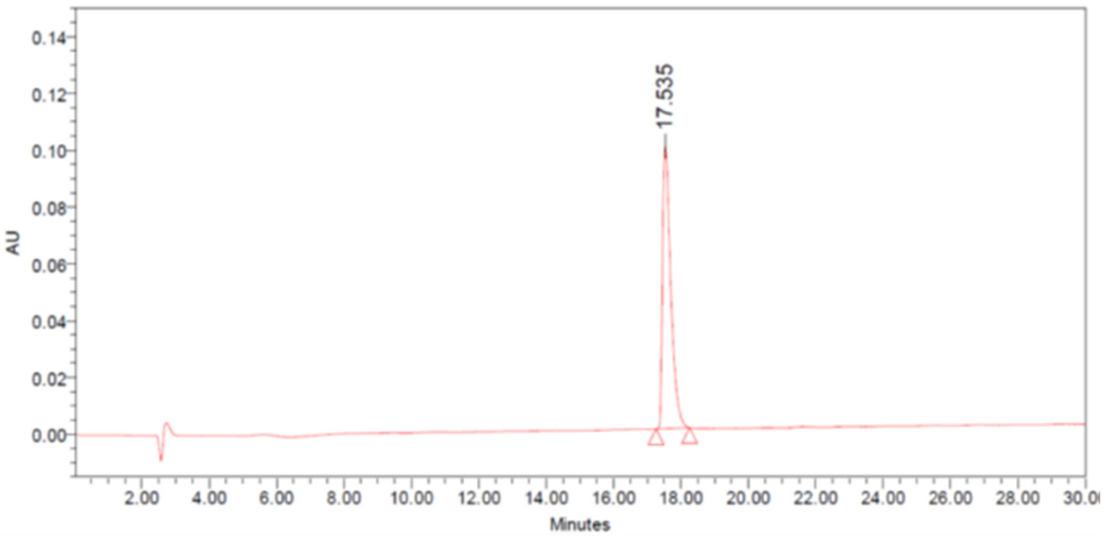

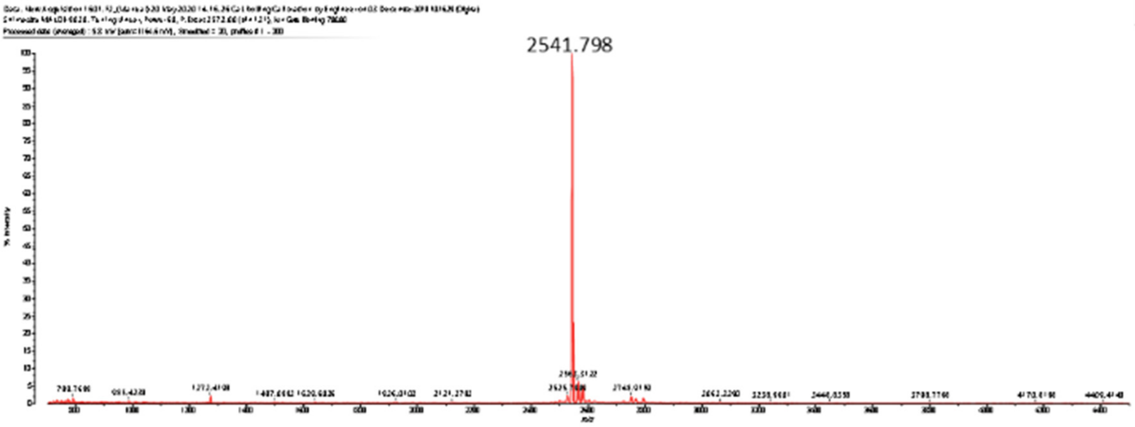

l. B9-31Aib11/18/25/29

RP-HPLC carried out using the elution gradient: buffer B 20-50% in 30 minutes, t= 17.535.

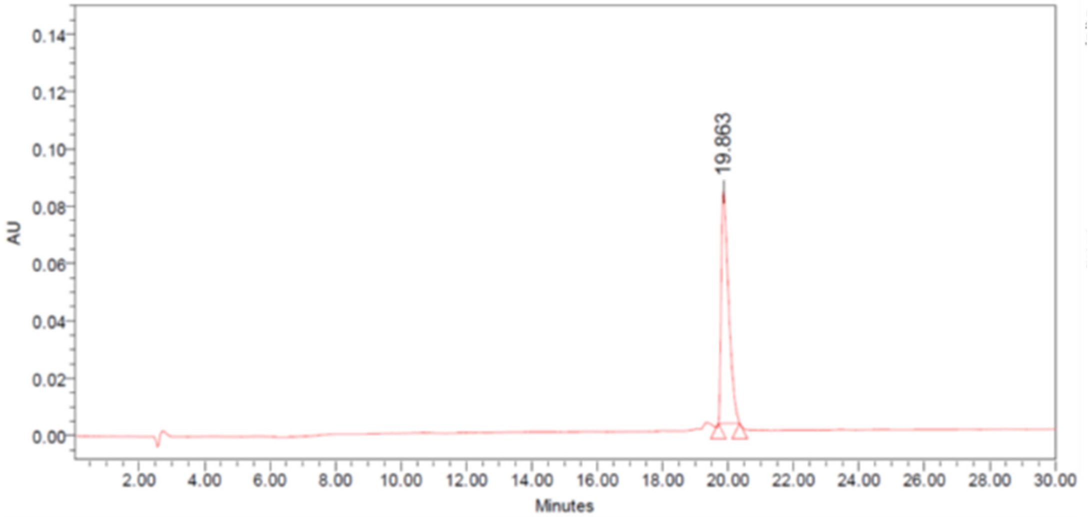

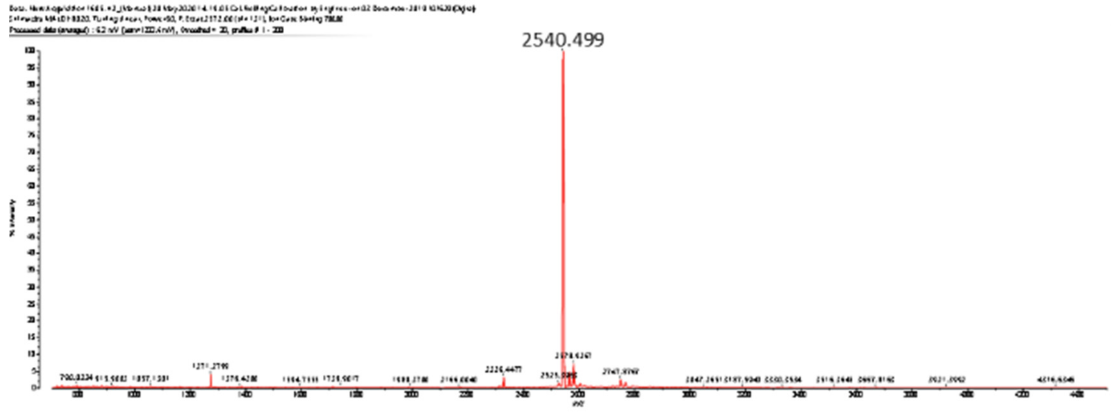

m. B7-33 S10-14
RP-HPLC carried out using the elution gradient: buffer B 25-55% in 30 minutes, t= 16.237.

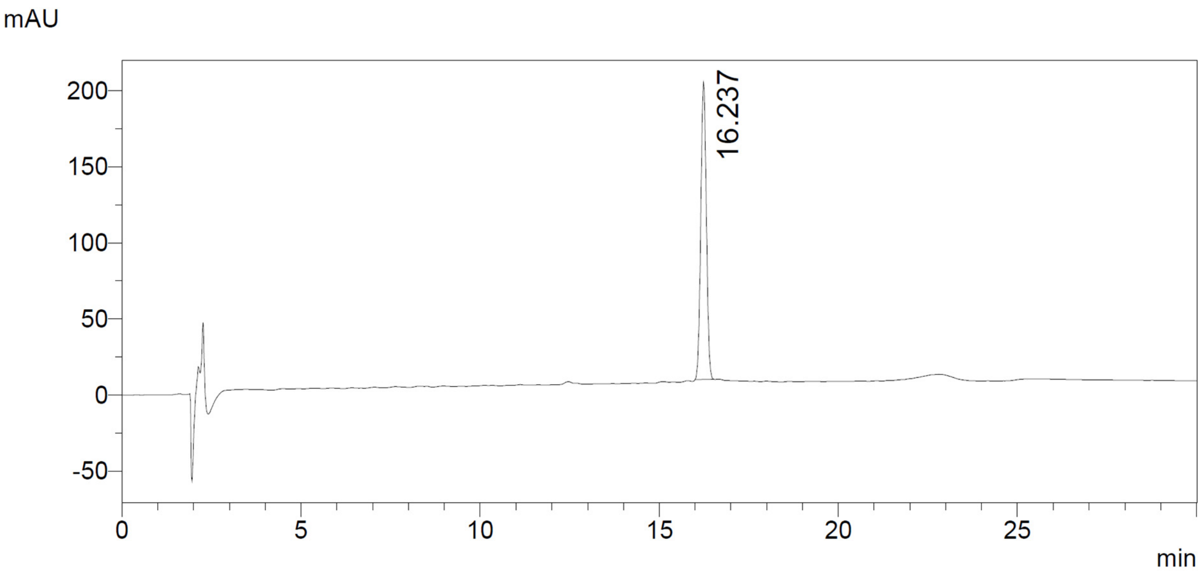

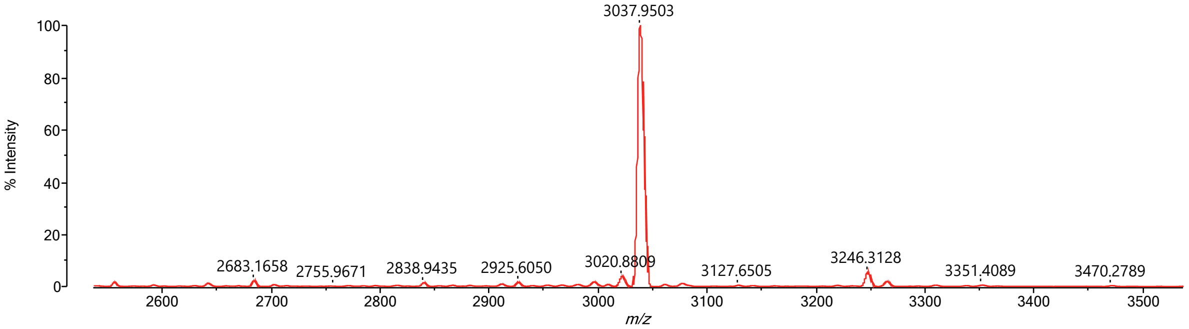

n. B7-33 S14-18
RP-HPLC carried out using the elution gradient: buffer B 25-55% in 30 minutes, t= 16.493.

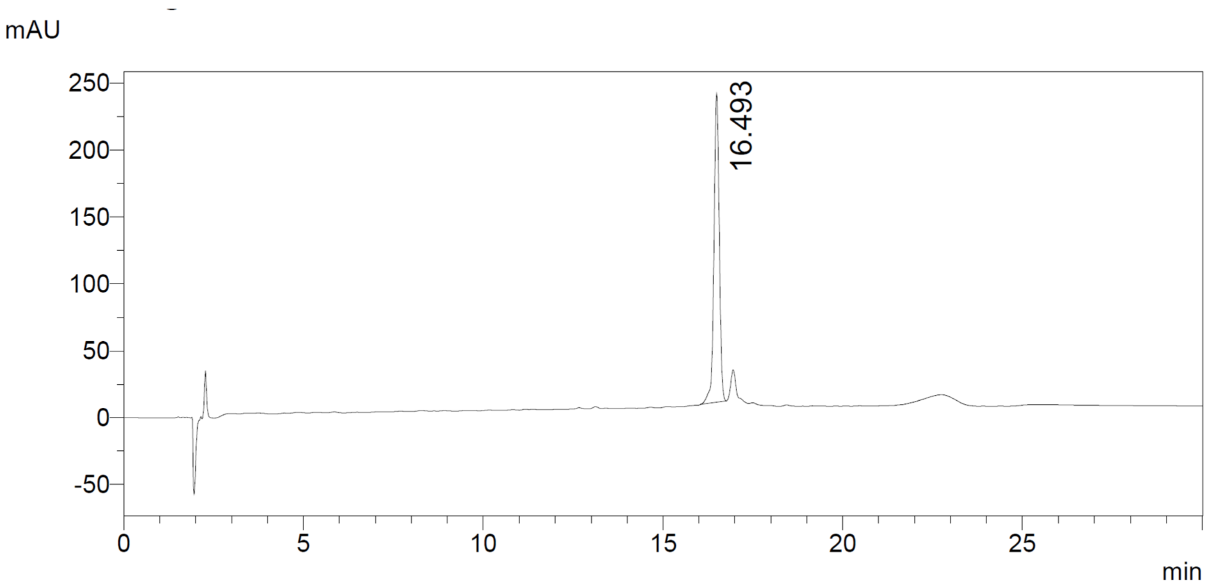

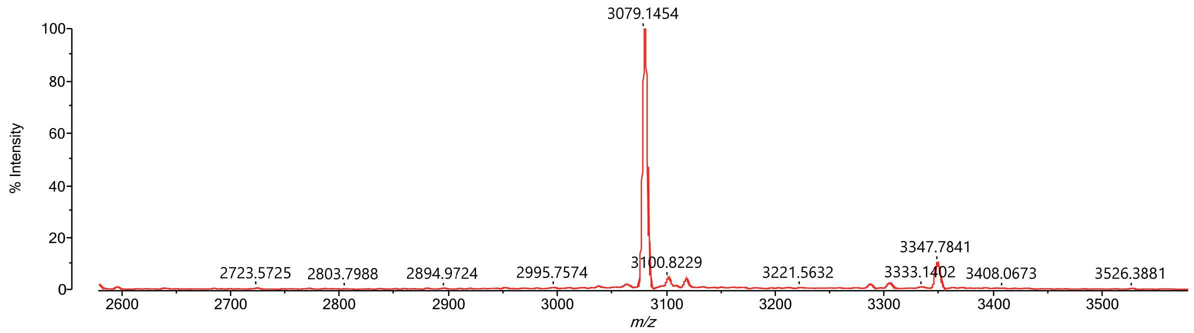

0. B7-33 S22-26
RP-HPLC carried out using the elution gradient: buffer B 25-55% in 30 minutes, t= 14.340.

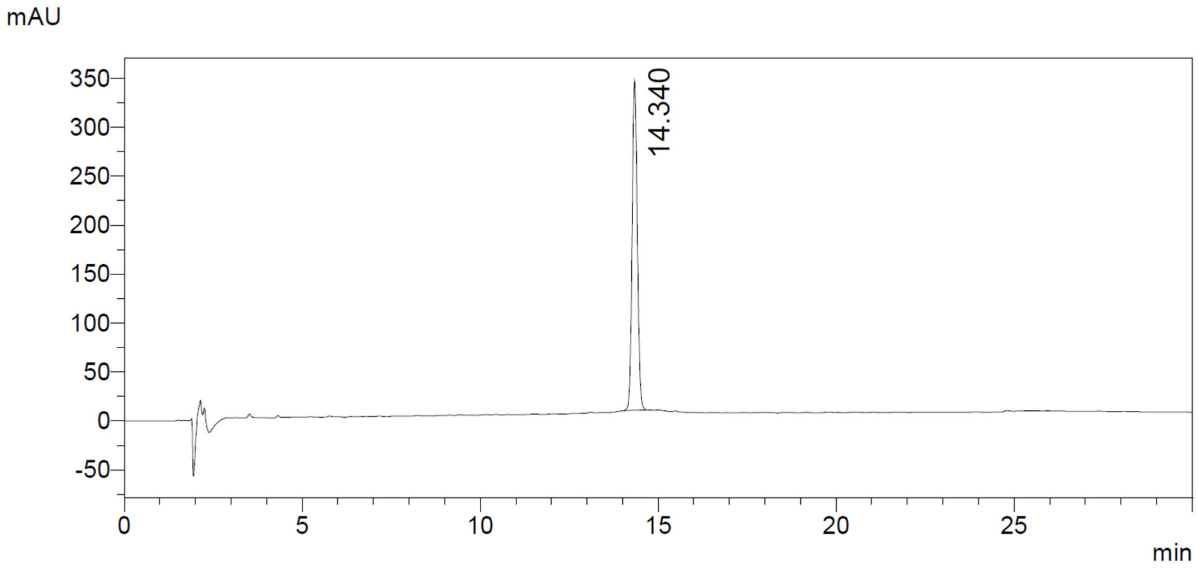

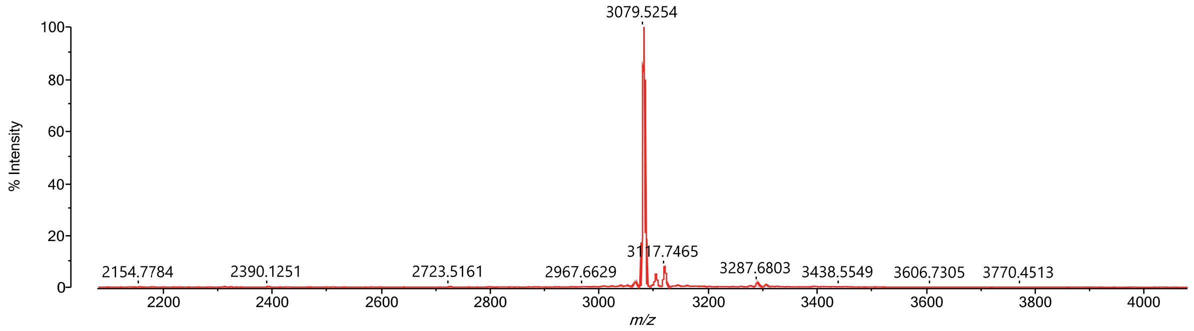

Supplement: Supplementary file 1 [file ijms-24-12670-s001.zip › ijms-2524231-supplementary.pdf]
